# Selectively Targeting of Gardeners and Symbiotic Fungus in Leaf-Cutting Ant Colonies Using Essential Oils

**DOI:** 10.3390/insects17060645

**Published:** 2026-06-18

**Authors:** Andressa Graebin, Patrícia F. Pinheiro, Karina D. Amaral, Vinicius F. Santos, Tarciza F. Nascimento, Marcela V. de S. Vilela, Yenara K. M. Silva, Thais D. Marcelino, Raul Narciso C. Guedes

**Affiliations:** 1 Departamento de Entomologia, Universidade Federal de Viçosa, Viçosa 36570-900, MG, Brazil; andressa.graebin@ufv.br (A.G.); karinadiamaral@gmail.com (K.D.A.); vinicius.f.santos@ufv.br (V.F.S.); 2 Departamento de Química, Universidade Federal de Viçosa, Viçosa 36570-900, MG, Brazil; patricia.pinheiro@ufv.br (P.F.P.); yenara.kendewry@gmail.com (Y.K.M.S.); 3 Departamento de Bioquímica e Biotecnologia, Universidade Federal de Viçosa, Viçosa 36570-900, MG, Brazil; tarciza.nascimento@ufv.br; 4 Departamento de Biologia, Universidade Federal de Viçosa, Viçosa 36570-900, MG, Brazil; marcela.vilela@ufv.br (M.V.d.S.V.); thais.marcelino@ufv.br (T.D.M.)

**Keywords:** leaf-cutting ants, toxic baits, caste-specific toxicity, fungal symbiosis, essential oils, *Atta sexdens*, *Acromyrmex subterraneus*

## Abstract

Social insect pests such as leaf-cutting ants challenge conventional pest management because effective control must disrupt colony-level organization rather than target individual insects. Colony persistence depends on the mutualistic association between gardener workers and their cultivated fungus, *Leucoagaricus gongylophorus*. Compounds that selectively impair these components while preserving forager-mediated bait transport may therefore offer strategic advantages. We evaluated the essential oils of weeping willow (*Salix babylonica*), Surinam cherry (*Eugenia uniflora*), weeping bottlebrush (*Melaleuca viminalis*), ginger (*Zingiber officinale*), and black pepper (*Piper nigrum*) against two leaf-cutting ant species, *Atta sexdens* and *Acromyrmex subterraneus*, after characterizing their chemical composition by GC–MS. The oils displayed distinct terpenoid profiles: bottlebrush oil, for instance, was dominated by 1,8-cineole and α-pinene, while ginger oil was rich in camphene and β-phellandrene. Forager and gardener workers were tested separately, along with their symbiotic fungus. Responses were generally concentration-dependent, although effects varied among oils and biological targets. Ginger oil exhibited strong fungicidal activity, but only at the highest concentration tested (100 mg mL^−1^). Bottlebrush oil showed marked toxicity to *A. sexdens* gardeners at concentrations as low as 0.10 mg mL^−1^, while effects on foragers were comparatively low. The remaining oils produced limited or inconsistent responses. These findings indicate that caste-selective toxicity and fungal suppression are achievable but not widespread among essential oils. Bottlebrush oil emerges as a promising candidate for further investigation, particularly regarding its constituent compounds and potential synergistic interactions for toxic bait development.

## Simple Summary

Leaf-cutting ants are major agricultural pests because they function as highly organized societies, which makes them difficult to control using methods that target individual insects. Therefore, control efforts should focus on limiting the worker ants responsible for feeding the cultivated fungus on which the colony depends. In this study, we tested natural plant extracts, known as essential oils, from five plant species to evaluate their effects on two leaf-cutting ant species and their cultivated fungus. We characterized these oils by gas chromatography–mass spectrometry (GC–MS), which revealed contrasting terpenoid profiles dominated by mono- and sesquiterpenes. We assessed whether these oils could harm the fungus or specific worker groups while allowing foraging ants to survive long enough to carry treated bait back to the nest. Most oils showed limited or inconsistent effects. However, ginger oil strongly inhibited fungal growth at high concentrations, and bottlebrush oil was particularly toxic to the workers responsible for maintaining the fungus, while having less effect on foragers. These findings suggest that some plant-derived compounds may help improve pest control strategies by targeting key components of ant colonies. Although further refinement is needed, such approaches could contribute to the development of more environmentally friendly pest management tools.

## 1. Introduction

Cooperative brood care, overlapping generations, and reproductive division of labor define eusociality, the highest level of social organization in animals [1,2]. In eusocial systems, colony persistence outweighs individual survival, and specialized castes collectively sustain reproduction, defense, and resource acquisition. Such organization confers ecological success, but also complicates pest management. In eusocial pests, control strategies must suppress the colony as a functional unit rather than eliminate isolated individuals.

Leaf-cutting ants exemplify this challenge. Distributed throughout the Neotropics, species of *Atta* and *Acromyrmex* constitute some of the most significant insect pests in agricultural and forest systems [3,4,5]. Since the early colonial period in Latin America, these ants have been regarded as a persistent and costly obstacle to crop and timber production [5]. Their economic impact derives not only from intense herbivory but also from the resilience, longevity, and structural complexity of their colonies.

Leaf-cutting ant colonies function as integrated biological systems centered on an obligate mutualism with the cultivated basidiomycete fungus *Leucoagaricus gongylophorus*. Leaf fragments collected by the worker ants are added to the fungal garden, where they are degraded to produce hyphal swellings, the gongylidia, which serve as the colony’s primary nutritional source. In addition to nutrient provisioning, the fungus contributes to the detoxification of plant defensive compounds that would otherwise impair colony performance [6,7]. Therefore, colony survival depends on the integrity of this ant–fungus association and on the minor workers, commonly referred to as gardeners, who maintain the fungal substrate [5,8,9]. Conquering such a biological “fortress” is not trivial. Management of leaf-cutting ants depends on suppressing the colony rather than killing exposed individuals [5].

Direct nest treatments are often impractical due to nest size and subterranean complexity. Consequently, control relies primarily on toxic baits composed of attractive organic substrates, typically citrus pulp pellets, impregnated with an insecticidal active ingredient, most commonly sulfluramid or fipronil [5,10]. Effective bait systems require a delicate balance: foraging workers must remain sufficiently functional to transport the bait to the nest, yet the active compound must ultimately impair key colony components, particularly the fungus garden and the minor workers that tend it [5,7,9,11]. Because the queen is deeply protected within the nest, suppression of gardeners and fungal symbionts constitutes the main pathway affecting queen and colony, leading to colony collapse [9,12].

Despite their efficacy, the active ingredients currently used in toxic baits face increasing regulatory and environmental scrutiny. Fipronil is associated with environmental persistence, soil contamination, and non-target toxicity [13,14], while sulfluramid may degrade into persistent perfluorinated compounds subject to international restriction under the Stockholm Convention [10,13,15,16]. Under certification frameworks such as those adopted by forest stewardship organizations, the continued use of these compounds often depends on derogation procedures [13,17]. As a result, forest-certified companies and integrated pest management programs increasingly seek alternative solutions with reduced environmental footprint, particularly within the realm of bioinsecticides [5,14,17].

Plants constitute a prolific source of biologically active secondary metabolites, evolved as part of their innate defense against herbivores and pathogens [18,19]. Among plant-derived products, essential oils are complex mixtures dominated by terpenoids, phenylpropanoids, and related volatile compounds whose biological activity reflects both individual constituents and potential synergistic interactions [20,21,22]. Essential oils have demonstrated insecticidal, antimicrobial, and antifungal properties across a range of target organisms, acting through contact, ingestion, or fumigation in insects, and through membrane disruption and interference with cellular integrity in fungi [23,24,25]. Such properties render them plausible candidates for incorporation into colony-targeted bait systems.

However, research evaluating essential oils against leaf-cutting ants remains comparatively limited and often focuses on worker mortality without distinguishing between castes or considering effects on the symbiotic fungus. Given that colony suppression depends primarily on the integrity of the fungal garden and its attending minor workers, assessing caste-specific responses and fungitoxic activity is therefore essential when evaluating plant-derived compounds for bait-based colony management.

We therefore selected five plant species with documented biological activity and distinct chemical profiles: weeping willow (*Salix babylonica*), Surinam cherry (*Eugenia uniflora*), weeping bottlebrush (*Melaleuca viminalis*), ginger (*Zingiber officinale*), and black pepper (*Piper nigrum)*. These species were chosen based on previous reports of insecticidal and/or antifungal activity of their extracts or essential oils against agricultural pests and phytopathogenic fungi, the presence of bioactive terpenoids and phenolics, and their local availability for extraction [26,27,28,29,30]. Despite this evidence, their effects on leaf-cutting ants and the mutualistic fungus *Leucoagaricus gongylophorus* remain largely unexplored, justifying their evaluation as candidates for colony-targeted control.

Two economically important leaf-cutting ant species were assessed: the parasol ant (*Atta sexdens*) and the quenquém (*Acromyrmex subterraneus*). Foragers and gardeners were tested separately, along with the cultivated fungus *L. gongylophorus*. We hypothesized that essential oil toxicity would vary with concentration and biological target, potentially revealing differential susceptibility among worker castes and measurable effects on fungal growth. We further hypothesized that selective impairment of gardeners and/or fungal development, coupled with comparatively lower toxicity to foragers, would represent a desirable profile for potential incorporation into toxic bait systems aimed at colony-level suppression. Thus, this study moves beyond conventional screening by integrating colony-functional targets with complex botanical oils.

## 2. Materials and Methods

### 2.1. Collection and Identification of Botanical Materials

Plant materials were collected in Minas Gerais and Espírito Santo States, Brazil, and voucher specimens were deposited in the Herbarium of the Federal University of Viçosa, where taxonomic identification was performed by specialists following standard herbarium procedures). Voucher codes were assigned as follows: weeping willow (*Salix babylonica*)–VIC58262; weeping bottlebrush (*Melaleuca viminalis*)–VIC58261; and Surinam cherry (*Eugenia uniflora*)–VIC58260. Ginger rhizomes (*Zingiber officinale*) were obtained commercially in Viçosa (MG, Brazil), and black pepper grains (*Piper nigrum*) were collected at Água Boa Farm, São Mateus, Espírito Santo, Brazil (18°4′36″ S, 40°02′49″ W).

Plant material was cleaned to remove debris, and leaves or rhizomes were chopped into small fragments immediately prior to hydrodistillation to minimize the loss of volatile constituents, following standard procedures for essential oil extraction.

### 2.2. Essential Oil Extraction and Yield

Fresh leaves of weeping willow (*S. babylonica*), Surinam cherry (*E. uniflora*) and weeping bottlebrush (*M. viminalis*), as well as ginger rhizomes (*Z. officinale*), were divided into three 100 g portions for hydrodistillation. For the black pepper, three 50 g portions of dried peppercorns were used. The samples were homogenized with 1 L of distilled water and subjected to hydrodistillation for 3 h using a modified Clevenger-type apparatus connected to a 2 L round-bottom flask, following established protocols for essential oil extraction carried out in triplicate [31].

The essential oils of Surinam cherry and ginger were collected by decantation. For the remaining species, the oils were recovered from the hydrosol (200 mL) by extraction with dichloromethane (3 × 15 mL). The combined organic phases were dried over anhydrous sodium sulfate, filtered, and concentrated under reduced pressure. Yields were determined gravimetrically, and the oils were stored in amber vials at −5 °C until use.

Chemical composition was determined by GC–MS (Shimadzu GCMS-QP2010C Ultra, Kyoto, Japan) equipped with an SPB-5 capillary column (30 m × 0.25 mm × 0.25 µm). Helium was used as carrier gas at 1.60 mL min^−1^. The oven temperature was programmed from 40 °C to 300 °C at 5 °C min^−1^. Injector and detector temperatures were 220 °C and 300 °C, respectively [32]. Compounds were identified by comparison of mass spectra with NIST library data and literature references, supported by calculation of linear retention indices (LRI) relative to n-alkane standards [33].

### 2.3. Ant Colonies

Colonies of the parasol ant (*Atta sexdens*) and the quenquém (*Acromyrmex subterraneus*) were collected in Viçosa, Minas Gerais, Brazil (20°45′14″ S, 42°52′54″ W) and maintained under controlled laboratory conditions (25 ± 5 °C; 75 ± 5% RH; 12:12 h L:D) as previously detailed [34]. Colonies were provisioned daily with acalypha leaves (*Acalypha wilkesiana*) and water, and they were maintained under controlled conditions of temperature (25 ± 5 °C), relative air humidity (75 ± 5%), and photoperiod (12 h:12 h, light/dark). Fungus gardens averaged approximately 1 L per colony. Colonies were deprived of fresh substrate for 24 h prior to bioassays to standardize physiological conditions [7].

### 2.4. Toxicity Bioassays with Worker Ants

Toxicity was evaluated separately for foraging (head capsule 2.0–2.2 mm) and gardening workers (0.8–1.0 mm) from five independent colonies per species. Topical application was employed as a standardized laboratory exposure method to ensure controlled dosing. Essential oils were diluted in 0.50% Tween 80 (Sigma-Aldrich, SP, Brazil) in distilled water at concentrations of 0.0001, 0.001, 0.01, 0.1, and 1 mg mL^−1^. The control consisted of 0.50% Tween 80 in distilled water. These concentrations were selected based on previous studies evaluating essential oils and terpenoid components against insect pests, and on preliminary range-finding assays aimed at spanning sublethal to clearly lethal exposure levels.

For each essential oil and caste, five replicates were used, each consisting of 10 workers and individuals originating from a single colony to preserve colony-level independence. Individuals were maintained in Petri dishes (10 cm diameter) with water and honey solution (1:1). Foraging workers received 1 µL of solution applied to the thorax; gardening workers received 0.5 µL. These volumes were chosen based on preliminary body-mass measurements and pilot assays, in which gardeners displayed approximately half the body mass of foragers, so that 0.5 µL in gardeners and 1 µL in foragers delivered a comparable dose per unit body mass without causing solvent-related mortality or obstructing spiracles and appendages. Controls received equivalent volumes of solvent solution. Mortality was recorded daily for seven days, and time-to-death (days) was used for survival analysis. Overall, concentration-dependent toxicity in quenquém is likely oil- and caste-specific, with clearer effects likely observed among gardeners than foragers, notwithstanding the use of body-mass-adjusted volumes to approximate similar dose per unit mass between castes.

### 2.5. Toxicity Bioassays with the Symbiotic Fungus

The fungus was isolated from nests of the parasol leafcutter ant by removing a small portion from the youngest part of the garden, a gray-colored region, and then aseptically transferring it to Petri dishes containing culture medium. The culture medium contained peptone, sodium chloride, malt extract, glucose, rolled oats, agar, distilled water, and rifampicin to prevent bacterial contamination. Media were autoclaved at 120 °C and 1.1 atm for 30 min [35].

The preliminary screening was conducted at 100 mg mL^−1^ (10 plates per essential oil and 10 control Petri dishes). The screening concentration and subsequent dilution series were chosen in line with published fungitoxicity assays of essential oils on phytopathogenic fungi and with our initial range-finding tests. Ginger and bottlebrush essential oils were subsequently evaluated at concentrations of 0.01, 0.1, 1, 10, and 100 mg mL^−1^, using 10 plates per concentration for each oil and 10 plates for the corresponding solvent control [36]. Oils were diluted in 0.50% Tween 80 in distilled water, and 100 µL were spread evenly onto the medium surface using a Drigalski loop to ensure uniform distribution. Controls received solvent only. A 1 cm-diameter fungal plug was placed at the center of each plate (10 mL of medium per plate). Plates were sealed and incubated at 25 ± 2 °C for 30 days. Fungal growth was quantified by measuring colony area using ImageJ 1.54 [37], and by determining final dry mass after oven-drying at 28 °C until constant weight.

### 2.6. Statistical Analyses

Survival analyses of both leaf-cutting ant species (*A. sexdens* and *Ac. subterraneus*) were performed separately for foraging and gardening castes exposed to different concentrations of essential oils. Time to death (days) was recorded for each individual, and survival curves were constructed using Kaplan–Meier estimators for each extract-concentration combination. Analyses were conducted in R using the survival, survminer, multcompView, and dplyr packages. The Surv() function was used to create survival objects from time and event (death or censoring) variables. Models were fitted using survfit(), stratified by concentration within each essential oil. Survival curves were plotted using ggsurvplot(), and global differences among concentrations were assessed using the log-rank (Peto–Peto) test [38]. When the global test was significant, pairwise log-rank comparisons among concentrations were performed, and *p*-values were adjusted using Bonferroni correction to control the family-wise error rate within each essential-oil × caste × species combination.

Fungal growth area and dry mass were initially assessed for normality (Shapiro–Wilk) and homogeneity of variances (Bartlett). As assumptions were not met and the Box–Cox transformation was unsuccessful, treatments were compared using the Kruskal–Wallis test, followed by multiple comparisons (*agricolae* package). For essential oils exhibiting concentration-dependent responses (ginger and bottlebrush), linear regression models were fitted to evaluate the relationship between concentration (log-transformed) and fungal growth parameters. Model assumptions were verified prior to interpretation. Results are presented as regression coefficients, R^2^, F statistics, and *p*-values.

## 3. Results

### 3.1. Chemical Composition, Identification, and Yield of Essential Oils

The chemical composition of the essential oils is presented in Table 1. The yield of essential oil from weeping willow leaves was 0.77% (*m*/*m*), with bicyclogermacrene (19.75%), spathulenol (18.29%), and myrcene (16.02%) as the major constituents. Surinam cherry leaves yielded 0.82% (*m*/*m*), with germacrone (36.57%), ledene (6.39%), and selina-3,7(11)-diene (2.89%) as the predominant components. In contrast, the essential oil yield from weeping bottlebrush leaves was lower (0.53% *m*/*m*) and was characterized by a strong predominance of 1,8-cineole (75.77%), followed by α-pinene (17.32%) and 3-cyclohexene-1-methanol, α,α,4-trimethyl (3.00%). The highest yields were obtained from ginger (1.82% *m*/*m*) and black pepper (1.40% *m*/*m*). Ginger essential oil was mainly composed of camphene (34.93%), β-phellandrene (27.91%), and α-pinene (11.86%), whereas black pepper essential oil was dominated by cis-caryophyllene (37.27%), limonene (21.41%), and β-pinene (12.67%).

### 3.2. Toxicity of Essential Oils to Quenquém Workers

**Figure 1:**
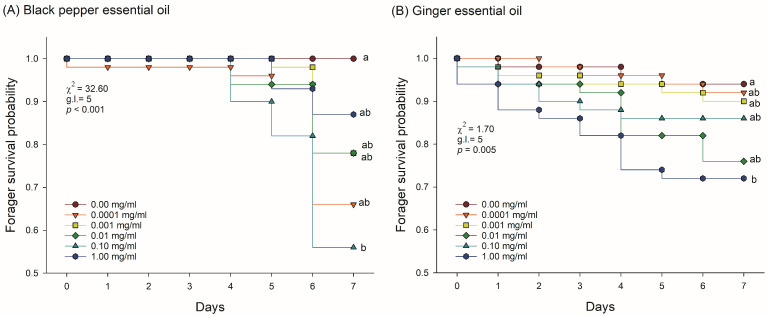
Kaplan–Meier survival curves showing the cumulative survival probability of quenquém forager ants (*Acromyrmex subterraneus*) following topical application of essential oils at concentrations of 0, 0.0001, 0.001, 0.01, 0.1, and 1 mg mL^−1^. Curves sharing the same letter do not differ significantly (*p* > 0.05).

**Figure 2:**
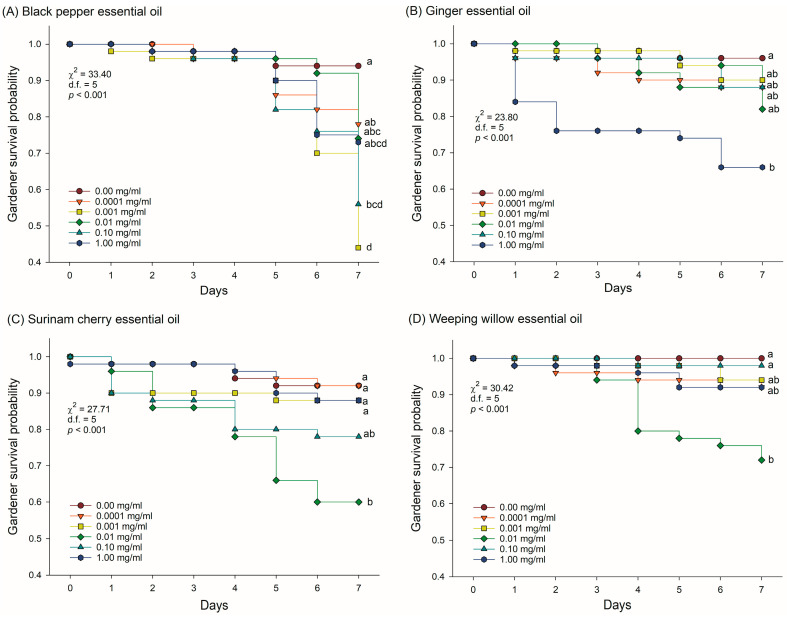
Kaplan–Meier survival curves showing the cumulative survival probability of quenquém gardener ants (*Acromyrmex subterraneus*) following topical application of essential oils at concentrations of 0, 0.0001, 0.001, 0.01, 0.1, and 1 mg mL^−1^. Curves sharing the same letter do not differ significantly (*p* > 0.05).

Among quenquém foragers, survival differed significantly across concentrations when exposed to black pepper and ginger essential oils (χ^2^ ≥ 32.60; d.f. = 5; *p* ≤ 0.005) (Figure 1). In contrast, no significant concentration-dependent effect was observed for weeping willow, weeping bottlebrush, or Surinam cherry essential oils (χ^2^ ≤ 8.97; d.f. = 5; *p* ≥ 0.11). For quenquém gardeners, significant differences in survival were detected for all essential oils (χ^2^ ≥ 23.8; d.f. = 5; *p* < 0.001), except for weeping bottlebrush (χ^2^ = 6.0; d.f. = 5; *p* = 0.31) (Figure 2). Overall, concentration-dependent toxicity in quenquém was oil- and caste-specific, with clearer effects observed among gardeners than foragers.

### 3.3. Toxicity of Essential Oils to Parasol Leafcutter Workers

**Figure 3:**
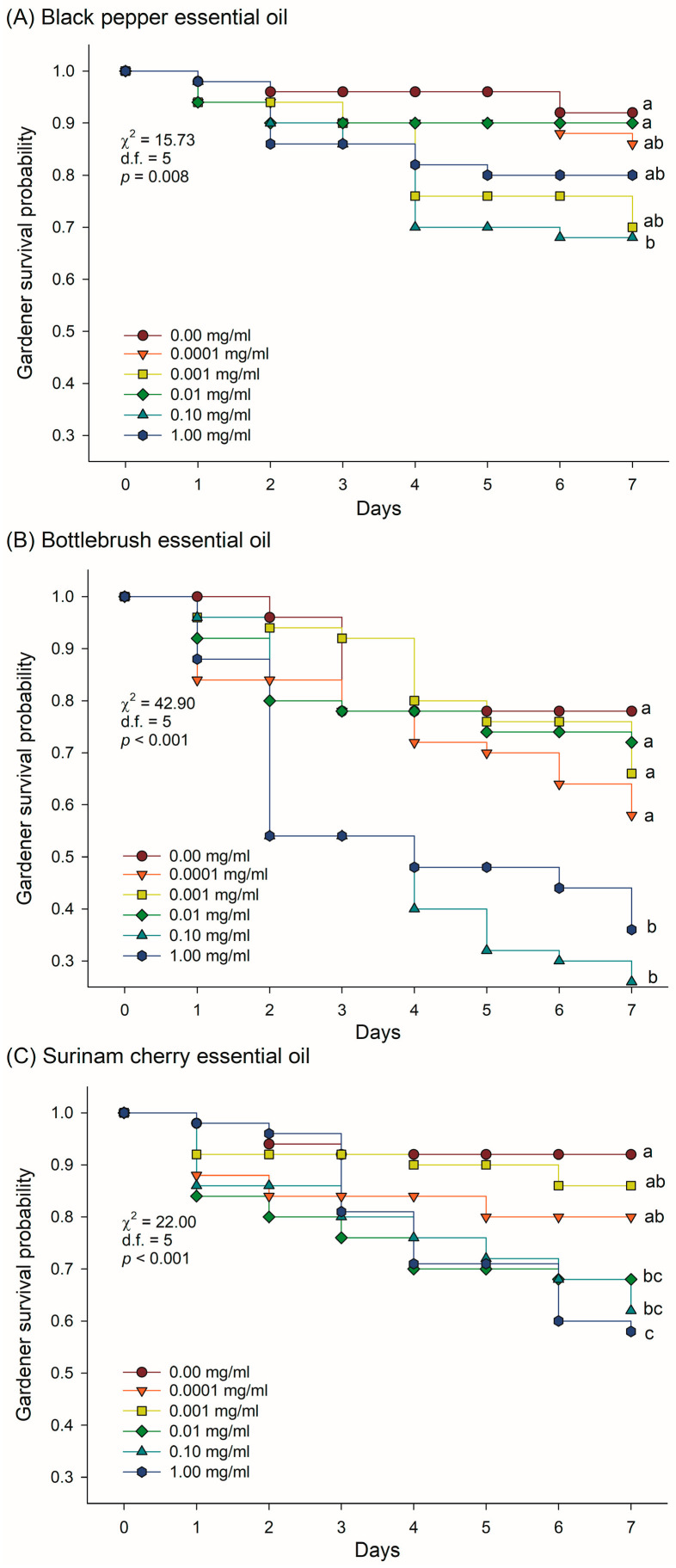
Kaplan–Meier survival curves showing the cumulative survival probability of parasol leafcutter worker ants (*Atta sexdens*) following in vitro topical application of essential oils at concentrations of 0, 0.0001, 0.001, 0.01, 0.1, and 1 mg mL^−1^. Curves sharing the same letter do not differ significantly (*p* > 0.05).

Among parasol leafcutter workers (*Atta sexdens*), no significant concentration-dependent effect was observed for foragers exposed to weeping willow or ginger essential oils (χ^2^ ≤ 5.52; d.f. = 5; *p* ≥ 0.36). Across treatments, no consistent concentration–mortality relationship was detected for leafcutter foragers (χ^2^ ≤ 8.70; d.f. = 5; *p* ≥ 0.12). In contrast, gardener survival differed significantly across concentrations for the essential oils of weeping bottlebrush, black pepper, and Surinam cherry (χ^2^ ≥ 15.7; d.f. = 5; *p* ≤ 0.008) (Figure 3). Across treatments, no consistent concentration–mortality relationship was detected for leafcutter foragers (χ^2^ ≤ 8.70; d.f. = 5; *p* ≥ 0.12). Thus, at the tested concentrations, essential oils exhibited limited toxicity toward leafcutter foragers, whereas bottlebrush, black pepper, and Surinam cherry oils showed insecticidal activity against gardeners.

### 3.4. Toxicity of Essential Oils to the Symbiotic Fungus L. gongylophorus

**Figure 4:**
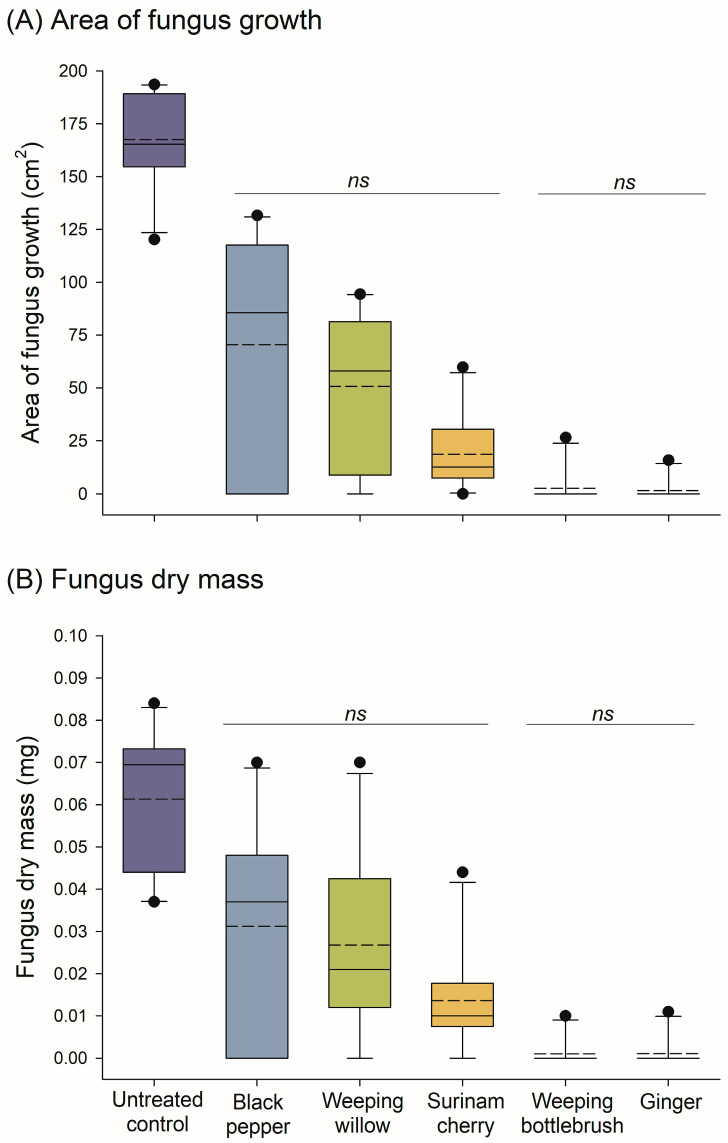
Effects of essential oils (100 mg mL^−1^) on (**A**) growth area (cm^2^) and (**B**) dry mass (g) of the symbiotic fungus *Leucoagaricus gongylophorus*. Comparisons among treatments were performed using the Kruskal–Wallis test (*p* < 0.05). Boxplots represent data distribution, with the horizontal line indicating the median; points beyond the whiskers are considered outliers. The notation “ns” indicates no significant difference among treatments.

In the control treatment, no inhibition of *L. gongylophorus* growth was observed. Control values were therefore used as the baseline to estimate inhibition in the essential oil treatments. The mean fungal growth area in the control was approximately 150 cm^2^, indicating normal development and differing significantly from treatments containing essential oils at 100.00 mg mL^−1^ (H = 20.18; d.f. = 5; *p* < 0.001) (Figure 4).

All essential oils significantly reduced fungal growth area and dry mass relative to the control (Figure 4). Bottlebrush and ginger essential oils completely suppressed mycelial development at 100.00 mg mL^−1^, resulting in visually undetectable growth and near-zero fungal biomass, consistent with strong fungicidal activity. Surinam cherry essential oil exhibited partial inhibition, with a mean growth area of approximately 20 cm^2^. In contrast, weeping willow and black pepper oils caused moderate reductions in growth, with mean areas of approximately 50 cm^2^ and 80 cm^2^, respectively (Figure 4).

**Figure 5:**
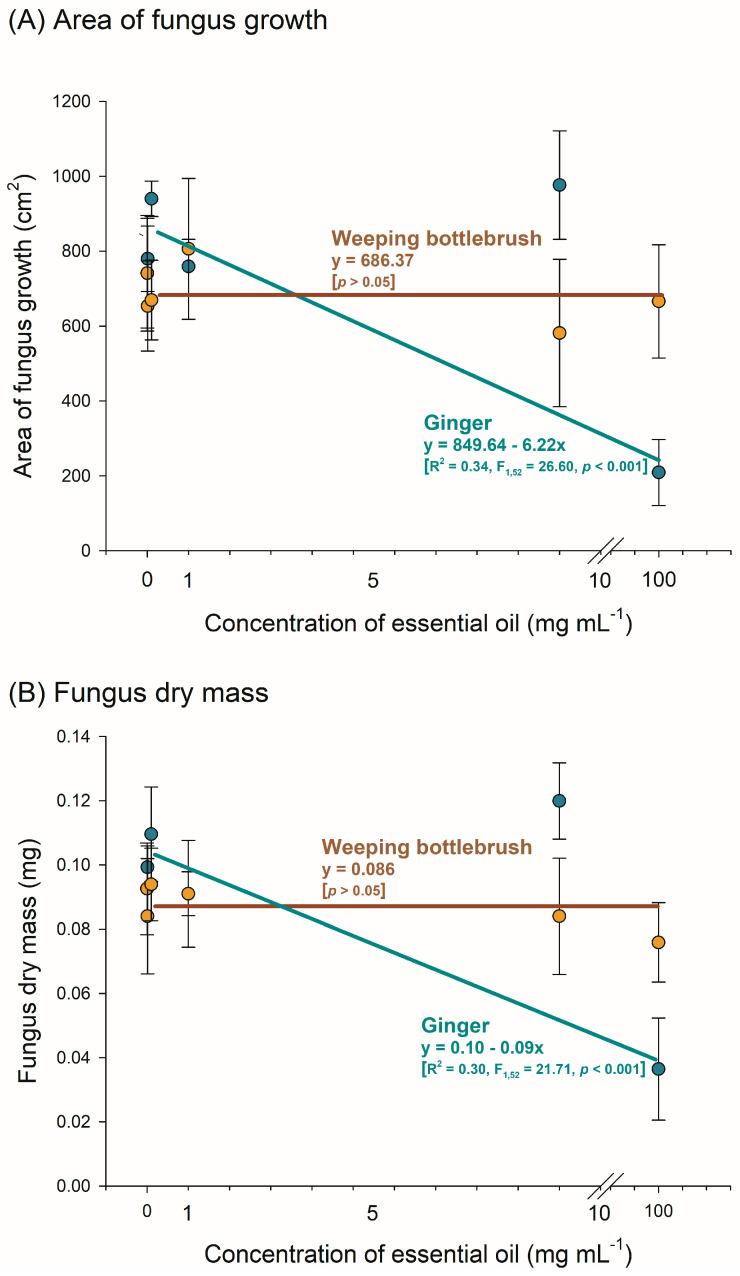
Effects of essential oils from ginger (*Zingiber officinale*) and weeping bottlebrush (*Melaleuca viminalis*) on (**A**) growth area (cm^2^) and (**B**) mass (g) of the symbiotic fungus *Leucoagaricus gongylophorus*.

Given their pronounced inhibitory effects at 100.00 mg mL^−1^, bottlebrush and ginger essential oils were further evaluated in concentration–response assays using lower concentrations up to the initial screening level. However, bottlebrush essential oil did not exhibit a significant concentration-dependent effect on fungal growth area or dry mass across the tested range (Figure 4). In contrast, ginger essential oil significantly reduced fungal growth area and dry mass at concentrations above 10.00 mg mL^−1^ (Figure 5).

## 4. Discussion

The growing interest in bioinsecticides reflects societal and regulatory concerns regarding the environmental persistence and non-target effects of conventional synthetic pesticides [21,39]. However, the distinction between ‘natural’ and ‘synthetic’ does not equate to safety, and both require careful ecological scrutiny [40,41,42]. While stored-product and commodity pests dominate the bioinsecticide literature, comparatively little attention has been directed toward social insect pests such as leaf-cutting ants [43]. Consequently, progress in developing effective ant baits based on bioinsecticides remains limited, with only a few preliminary candidates showing potential [8,12,44,45].

The design of toxic ant baits imposes specific biological constraints. Effective compounds must exhibit low toxicity toward foragers—so as not to prevent bait transport to the nest—while affecting gardeners, queens, or the symbiotic fungus responsible for colony nutrition [5,8,9]. Additionally, high vapor pressure, rapid degradation, or excessive acute toxicity may impair colony-level suppression; for example, overly fast-acting compounds can kill foragers before they return to the nest, reducing bait intake and limiting downstream effects on gardeners and the fungus [5,8]. These constraints partly explain the continued reliance on sulfluramid and fipronil, despite regulatory restrictions and derogations [10].

In this context, we evaluated the bioinsecticidal potential of essential oils from five plant species against two worker castes, foragers and gardeners, of two leaf-cutting ant species, and against their symbiotic fungus *Leucoagaricus gongylophorus*. We hypothesized that toxicity would be caste-dependent and concentration-dependent, and that fungal growth would be susceptible to oil exposure. Our results partially support these hypotheses.

Caste-specific responses were evident. Concentration-dependent effects were more pronounced among gardener workers than foragers, particularly for black pepper and Surinam cherry essential oils. Ginger and willow oils showed concentration-dependent responses only in quenquém gardeners, whereas bottlebrush oil affected *Atta sexdens* gardeners. Importantly, forager mortality was generally low, a desirable characteristic for bait-based delivery systems. However, gardener mortality rarely exceeded 50% after seven days, which falls below the efficacy typically required for registration of conventional insecticides in Brazil [46]. Thus, although selective activity toward gardeners was observed, the magnitude of insecticidal effects was limited. Nonetheless, even partial suppression of gardener workers may increase fungal vulnerability by reducing maintenance and hygiene behaviors, potentially delaying colony destabilization.

The essential oils evaluated here were dominated by mono- and sesquiterpenes, including α- and β-pinene, limonene, 1,8-cineole, bicyclogermacrene, germacrone, and caryophyllene derivatives. Many of these compounds have been associated with insecticidal, repellent, or fungitoxic activity, acting through neurotoxicity, membrane disruption, and interference with cellular integrity [22,25,26,27]. For example, camphene, germacrone, and α-humulene have shown significant toxicity towards Lepidopteran and mite pests, while monoterpenes such as limonene and 1,8-cineole can impair both insects and fungi [47,48].

The relative peak area profiles in Table 1 suggest that oils enriched in monoterpenes, as in ginger and bottlebrush, may favor stronger acute or fungicidal effects, whereas oils with higher proportions of sesquiterpenes, such as Surinam cherry, displayed more moderate or inconsistent responses in our assays [20,21,23,24,25,47,48,49]. At the same time, the biological activity of essential oils arises from the combined action of major and minor constituents, including synergistic and antagonistic interactions [50], so our GC–MS data should be viewed as indicative rather than strictly predictive of individual compound effects [20,21,22,50]. The relatively modest toxicity observed here suggests that optimization through fractionation, formulation, or synergistic combinations would likely be required to enhance activity toward target castes.

Within this framework, some major constituents emerge as likely contributors to the observed activity. Bottlebrush oil contained high levels of 1,8-cineole and α-pinene, which have been frequently implicated in insecticidal and fungitoxic effects. Ginger oil was enriched in camphene and β-phellandrene, compounds that have shown toxicity to lepidopteran larvae and stored-product pests, whereas black pepper oil was dominated by cis-caryophyllene and limonene, both associated with insecticidal or repellent properties in other systems. The stronger fungicidal effect of ginger oil and the caste-biased toxicity of bottlebrush oil are therefore consistent with the presence of these active terpenoids, although the complex mixture and potential interactions among constituents preclude definitive attribution to any single compound [19,20,21,45].

At the mechanistic level, monoterpenes and sesquiterpenes can act on insects by disrupting cuticular and cellular membranes, altering ion gradients, inhibiting acetylcholinesterase and other neural enzymes, and inducing oxidative stress [20,22,25]. In phytopathogenic fungi, similar compounds interfere with ergosterol biosynthesis and membrane integrity, leading to leakage of intracellular constituents and impaired growth [25,26,50]. Numerous studies also indicate that combinations of terpenoids can exhibit synergistic or antagonistic interactions, so that the efficacy of an essential oil often exceeds what would be predicted from its major components alone [51]. The moderate but selective effects observed here are therefore likely to arise from both the activity of individual compounds and their interactions within each oil.

An alternative and potentially more promising target for ant bait development is the symbiotic fungus *L. gongylophorus*, which sustains colony growth [6]. Essential oils are known to disrupt fungal membrane integrity, interfere with ergosterol biosynthesis, and alter cell permeability [20,23]. In our study, only bottlebrush and ginger essential oils completely suppressed fungal growth at 100 mg mL^−1^. However, when tested across a broader concentration range, only ginger oil maintained a significant concentration-dependent effect, but only at higher doses.

The discrepancy observed for bottlebrush oil between the initial screening and subsequent concentration-response assays may reflect compositional instability or volatility-driven changes during storage and handling. As shown in Table 1, bottlebrush oil is dominated by the monoterpenes 1,8-cineole and α-pinene, which are relatively volatile and susceptible to oxidative degradation and evaporative loss during storage, heating, or repeated handling. A reduction in the proportion or absolute amount of these major constituents between the screening and follow-up assays could plausibly attenuate fungicidal potency, particularly at lower concentrations.

Essential oil composition is known to vary with extraction method, storage conditions, temperature, light exposure, and plant phenological stage [25]. Such variability can directly influence biological activity and highlights the need to monitor chemical stability in bioassay-driven studies. At the same time, we cannot exclude contributions from experimental variation, including differences among fungal subcultures and plate-to-plate variability, to the inconsistent bottlebrush response across assays.

Overall, ginger essential oil demonstrated fungicidal activity at high concentrations and limited caste-selective insecticidal effects, whereas bottlebrush oil exhibited moderate gardener toxicity and strong fungal inhibition at the screening concentration. Similar to other studies with plant-derived products against social insects, the efficacy observed here is lower than that of established synthetic active ingredients such as sulfluramid and fipronil, which still underpin most operational bait formulations despite regulatory constraints. This reinforces the notion that essential oils alone are unlikely to provide complete and reliable suppression of mature leaf-cutting ant colonies.

Given the biological complexity and resilience of leaf-cutting ant societies, practical control strategies will likely require multi-target approaches that disrupt both worker castes and the symbiotic fungus, and that integrate different modes of action [5,8,9,11,12]. In this context, essential oils and their purified constituents may be better positioned as components of integrated bait systems—either as co-active ingredients, synergists, or fungitoxic additives—rather than stand-alone replacements for current insecticides [19,20,21,22,45,50]. Our findings thus highlight the importance of targeting functionally distinct components of social insect colonies and provide initial guidance for selecting chemical profiles that merit further optimization through formulation, fractionation, and synergistic combinations.

## Figures and Tables

**Table 1:** Chemical characterization of the essential oils from weeping willow (*Salix babylonica*), Surinam cherry (*Eugenia uniflora*), weeping bottlebrush (*Melaleuca viminalis*), ginger (*Zingiber officinale*), and black pepper (*Piper nigrum*).

	Contituents	AI (calc.)	AI (lit.)	Relative Peak Area (%)				
				Weeping Willow *(S. babylonica)*	Surinam Cherry *(E. uniflora)*	Weeping Bottlebrush *(M. viminalis)*	Ginger *(Z. officinale)*	Black Pepper *(P. nigrum)*
1	α-Thujene	942	924	-	-	-	-	0.55
2	Camphene	945	946	-	-	-	35.17	-
3	α-Pinene	948	932	0.95	-	17.32	11.95	5.71
4	Sabinene	989	969	13.23	-	-	-	4.76
5	β-Pinene	991	974	-	-	1.74	-	12.67
6	α-Phellandrene	1003	1002	-	-	-	1.04	-
7	Myrcene	1008	988	-	-	0.40	2.72	1.58
8	β-Myrcene	1008	NI	16.02	-	-	-	-
9	β- Phellandrene	1027	1025	0.37	-	-	28.11	-
10	1,8-Cineole	1029	1026	-	-	75.77	5.72	-
11	Propanoic acid, 2-methyl, pentyl ester	1030	1049	-	-	1.23	-	-
12	α-Terpinene	1032	1014	0.12	-	-	-	-
13	Benzene, 1-methyl-2-(1-methylethyl)- (CAS) 1-Methyl-2-isopropylbenzene	1039	NI	0.17	-	-	-	-
14	Limonene	1044	1024	-	-			21.41
15	γ-Terpinene	1074	1054	0.23	-	0.54	-	-
16	Furan, 3-(4-methyl-3-pentenyl)- (CAS) Perillen	1117	1102	0.46	-	-	-	-
17	3-cyclohexene-1-methanol, α,α,4-trimethyl	1206	1186	-	-	3.00	-	-
18	3,7-Nonadien-2-one, 8-methyl	1260	NI	-	-	-	1.26	-
19	α-Copaene	1392	1374	-	-	-	-	6.24
20	(-)-Elema-1,3,11(13)-trien-12-ol	1410	-	-	0.45	-	-	-
21	Trans-caryophyllene	1437	1417	13.47	1.45	-	-	-
22	cis-caryophyllene	1438	1408	-	-	-	-	37.27
23	α-Amorphene	1457	1483	-	0.85	-	-	
24	α-Humulene	1470	1452	0.99	-	-	-	2.34
25	α-Zingiberene	1480	1493	-	-	-	10.04	-
26	Farnesene <(E,E)-α->	1492	1505	-	-	-	2.51	-
27	Germacrene D	1498	1480	7.22	-	-	-	-
28	Trans-β-Guaiene	1505	1502	-	0.25	-	-	-
29	β-Sesquiphellandrene	1507	1521	-	-	-	1.49	-
30	Ledene	1515	1496	-	6.39	-	-	-
31	Bicyclogermacrene	1515	1500	19.75	-	-	-	-
32	Curzerene	1516	1499	-	1.36	-	-	-
33	β-Bisabolene	1525	1505	-	-	-	-	3.29
34	δ-Cadinene	1540	1522	-	-	-	-	1.81
35	NI	1555	1452	-	4.77	-	-	-
36	Selina-3,7(11)-diene	1562	1545	-	2.89	-	-	-
37	Spathulenol	1596	1577	18.29	-	-	-	-
38	Caryophyllene oxide	1600	1582	8.73	-	-	-	2.36
39	Germacrone	1718	1693	-	36.57	-	-	-
40	NI	1775	1476	-	24.44	-	-	-
41	NI	1779	1476	-	20.58	-	-	-

TR = retention time; AI (calc.) = calculated arithmetic index; AI (lit.) = literature arithmetic index; %Area = relative peak area calculated from the chromatogram; NI = not identified.

## Data Availability

The datasets generated during and/or analyzed during the current study are available from the corresponding author on reasonable request.
